# Economic impact of nature-based tourism

**DOI:** 10.1371/journal.pone.0282912

**Published:** 2023-04-12

**Authors:** Anubhab Gupta, Heng Zhu, Hasita Bhammar, Elisabeth Earley, Mateusz Filipski, Urvashi Narain, Phoebe Spencer, Edward Whitney, J. Edward Taylor

**Affiliations:** 1 Department of Agricultural and Applied Economics, Virginia Tech, Blacksburg, VA, United States of America; 2 World Food Programme, Kampala, Uganda; 3 The World Bank, Washington, DC, United States of America; 4 Department of Political Science, University of California, Berkeley, Berkeley, CA, United States of America; 5 Department of Agricultural & Applied Economics, University of Georgia, Athens, GA, United States of America; 6 Department of Agricultural & Resource Economics, University of California, Davis, Davis, CA, United States of America; University of Sargodha, PAKISTAN

## Abstract

Protected areas (PAs) can help address biodiversity loss by promoting conservation while fostering economic development through sustainable tourism. Nature-based tourism can generate economic benefits for communities in and around PAs; however, its impacts do not lend themselves to conventional impact evaluation tools. We utilize a Monte Carlo simulation approach with econometric estimations using microdata to estimate the full economic impact of nature-based tourism on the economies surrounding three terrestrial and two marine PAs. Simulations suggest that nature-based tourism creates significant economic benefits for communities around PAs, including the poorest households, and many of these benefits are indirect, via income and production spillovers. An additional tourist increases annual real income in communities near the PAs by US$169—$2,400, significantly more than the average tourist’s expenditure. Conversely, lost tourism due to the COVID-19 pandemic and economic costs of human-wildlife conflict have disproportionately large negative impacts on local incomes.

## Introduction

Since 1970, one-third of the world’s terrestrial and two-thirds of the world’s marine ecosystems have been rapidly degrading due to loss of habitat, climate change, and pollution, causing catastrophic biodiversity loss [1]. Biodiversity matters not only because of its intrinsic worth to every living species, but also because ecosystem-based services such as nature-based tourism, which depend on biodiversity, can promote human wellbeing, help reduce poverty, and create economic incentives for local populations to protect the resource [2–4].

The Convention on Biological Diversity Aichi Biodiversity Target 11 calls for the conservation of “at least 17% of terrestrial and inland water areas and 10% of coastal and marine areas, especially areas of particular importance for biodiversity and ecosystem services,” through “effectively and equitably managed, ecologically representative and well-connected systems of protected areas and other effective area-based conservation measures, and integrated into the wider landscapes and seascapes.” As of June 2022, roughly 253,359 terrestrial and inland waters PAs covered approximately 15.8% of all terrestrial and inland water areas, and 17,781 marine protected areas covered 8.13% of the world’s ocean area [5].

PAs around the world attract approximately 8 billion visits in a typical year [6], but the COVID-19 pandemic severely disrupted both international and domestic tourism. International tourism contracted by 74% between January and December 2020 (reduction of about 1 billion trips), causing an estimated US$4 trillion loss globally, with a greater burden on developing economies [7, 8]. Tourism to PAs in the post-pandemic era could help promote biodiversity conservation while contributing towards rebuilding poor economies ravaged by the COVID-19 pandemic.

The creation and maintenance of PAs face on-going challenges including poor management, illegal wildlife trade, competition over natural resources, human-wildlife conflict, and lack of finance and community engagement. An assessment of coral reefs in marine PAs of the Pacific Ocean’s Coral Triangle found that only 1 percent of these areas were effectively managed [9]. Poor management of terrestrial PAs contributes to deforestation, which may lead to a loss of formal protection through downsizing or degazetting [10, 11]. Lack of management plans, equipment, infrastructure, size, and designation of PAs also adversely affect conservation outcomes [12]. There is evidence of increased poaching and exploitation of natural resources in terrestrial PAs in Asia, southern and eastern Africa, and marine PAs globally due to poor enforcement [13, 14]. These problems may have increased due to park closures and funding cutbacks during the pandemic [15]. Competition over natural resources intensifies the challenges to PA management. In Latin America, large-scale habitat loss from agricultural expansion, infrastructure development, cattle ranching and fires threaten fragile ecosystems. In sub-Saharan Africa, cropland coverage inside PAs has increased at a rate nearly double the rate in non-PAs. Outside the Amazon biome, agricultural pressure in PAs increased by 10% over the last 15 years [16].

A biodiversity funding gap, estimated at between US$598 billion and US$824 billion per year, constrains the effective management of PAs [17]. PAs are underfunded worldwide [18–22]. Nearly all PAs in Africa are inadequately funded, and a deficit of US$1 billion annually must be addressed to save iconic species and landscapes there [23]. PAs in Latin America are under-funded by approximately US$700 million annually [24]. The funding needed for a global network of marine PAs covering 20–30% of the seas is estimated to be between US$5 and US$19 billion per year [25].

Lack of funding for PAs reflects a widespread perception that biodiversity conservation competes with economic development and the economic costs of preserving natural environments outweigh the benefits. Incomplete accounting for economic benefits from PAs contributes to this perception. PAs, including national parks and other types of terrestrial and marine preserves, are usually found in relatively remote and neglected, but biologically rich, rural regions. Economic incentives for conservation are important considering that protecting natural environments entails economic costs, including those associated with human-wildlife conflict [26–28].

A unique collaboration between the World Bank and researchers from several universities in the US and abroad examined the economic impacts of tourism on the economies near two marine and three terrestrial PAs in four countries—Brazil, Fiji, Nepal, and Zambia. The findings from this study provide evidence that promoting sustainable and inclusive nature-based tourism creates direct and indirect economic benefits for communities adjacent to PAs, including for poor households that may rarely interact directly with tourists. This study reveals heretofore under-appreciated economic impacts of PAs through sustainable tourism.

The rest of the paper is organized as follows: the following subsection provides a brief review of the existing literature, the section on materials and methods describes the methodology, data, econometric estimations, and simulations, the next section discusses the results on the economic impact of tourism, and the final section concludes with policy discussions.

### Review of existing literature

Previous research on tourism impacts used input-output or social accounting matrix (SAM) based models, coupled with tourist expenditure surveys, to estimate value-added impacts [29, 30]. Other studies have shown the impact of tourism spending and tourism market diversification on economic growth and environmental pollution using time-series models in several countries [31–33]. However, most studies only capture direct impacts of tourism, for example, tourist spending on park fees or at hotels, restaurants, and other businesses that cater to tourists. Tourist spending represents only the first-round impact of tourism on local economies. Studies focusing on tourist spending fail to account for potentially large higher-order (indirect) impacts resulting from production and consumption linkages among households, businesses, and other institutions. For example, tourist expenditures benefit households that earn wages or profits from tourism-related activities. These households, in turn, spend money in non-tourism businesses, which generate income for other households and unleash additional rounds of increases in local demand. Fixed-price multiplier models seek to capture these multiple rounds of impacts. However, they tend to overstate real income and production impacts by not considering local price effects that influence the supply of goods and services, including production factors like labor. If the supply does not increase in tandem with increased local demand, tourist spending may cause price inflation while reducing real economic growth. In this paper, we are able to estimate the secondary-level effects of tourism in PAs through a local economywide general equilibrium model that connects various household groups in the local economies of PAs through inputs, production, and consumption market linkages.

**Fig 1:**
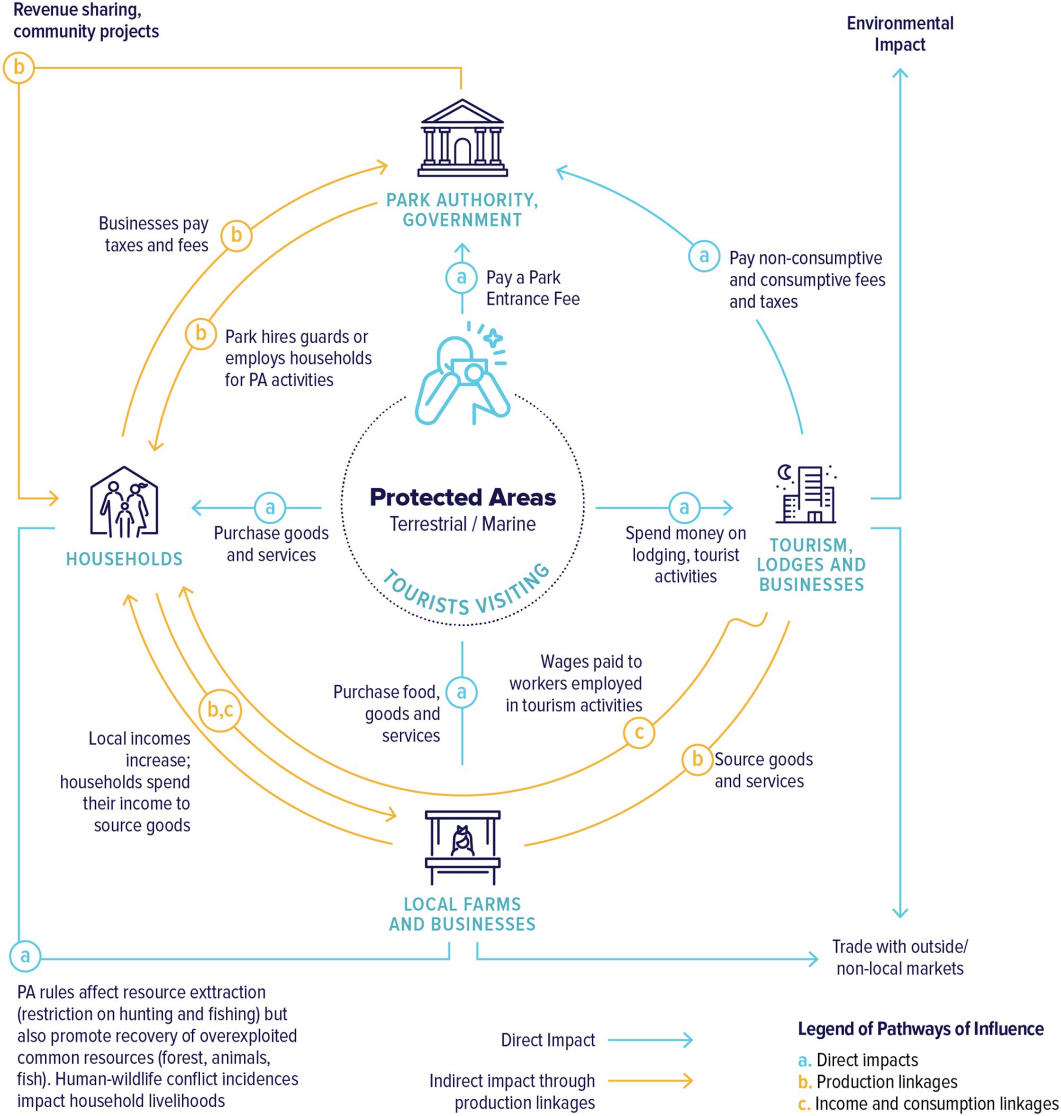
Economic impact pathways for protected areas. The inner channels “a” (in light blue color) show the direct impacts through tourist spending on park entrance fees, which accrue to park authorities/government; lodging and tourism activities, provided by tourism lodges and local businesses; and goods and services supplied by local households, farms, and businesses. The outer circles “b” and “c” (in orange) describe the production, income, and consumption linkages within the local economy near Protected Areas.

Tourism to PAs stimulates economic activities that cater to tourists. As these activities expand, so do their demands for goods and services, which may be sourced inside or outside the local economy. Direct contributions to the economy include visitor spending on park fees, hotels, transport, leisure and recreation, which create employment and support local businesses. Indirect effects occur when tourism businesses generate profits and wages for households, and both the businesses and households increase their demand for locally supplied goods and services. Multiple rounds of increased production, household incomes, and expenditures contribute to local real income multipliers from PA tourism, defined as changes in total household real (inflation-adjusted) income per unit of income injected into the local economy by tourists (see Fig 1).

This study uses new micro-survey data collected just before the onset of the COVID-19 pandemic from households, local businesses, hotels, and tourists at three terrestrial and two marine PAs: Lower Zambezi and South Luangwa National Parks in Zambia (terrestrial); Chitwan National Park in Nepal (terrestrial); Abrolhos Marine Park in Brazil (marine) and Fiji’s Mamanuca Islands (marine). The sites chosen for the study represent a mixture of economies, geographies, and culture. The four countries all have existing World Bank engagements focusing on PAs and tourism. PAs within the countries were chosen through consultations with governments. Local economies consist of the communities lying within the PA’s sphere of economic influence, as determined by community members, tourism operators, and the government. Because village households and businesses routinely visited a nearby market town to purchase goods and services, the market town nearest each PA were included as part of the local economy. (S1 Text in S1 Appendix outlines the criteria used to define the local economy at each study site.)

We estimated impacts of PA tourism on the local economy around each site in two steps. First, we use microdata from the surveys to calibrate an economy-wide model to simulate the impact of an *additional tourist* and of an *additional dollar of tourist spending* on each local economy and calculate income multipliers. Secondly, we estimate *total tourism impacts* by multiplying the per-tourist multiplier by the total number of tourists visiting each site. There is no way to know what the true counterfactuals would be without tourism, but this approach provides the best approximation of the economic impacts of tourism in protected areas.

## Materials and methods

Analysis of economic impacts of nature-based tourism does not lend itself to conventional experimental approaches such as randomized control trials (RCTs), instrumental variable methods, and “quasi-natural” experiments, particularly given the non-random location of PAs. Baseline data before the creation of PAs are rare, and this limits the potential use of econometric methods to estimate PA impacts by attempting to emulate experiments. Applied general equilibrium (GE) methods are a promising approach to quantify both direct and indirect impacts of nature-based tourism on economies near PAs. Our simulation model uses a local economy-wide impact evaluation (LEWIE) approach (31), designed to understand the direct and indirect (or “spillover”) effects of tourism to PAs on local economies. We study five sites—three terrestrial PAs (two in Zambia and one in Nepal), and two marine PAs (one in Brazil and one in Fiji).

At each site, a series of micro-economic models of diverse household groups were constructed through econometric analysis of the survey data then integrated into a general equilibrium (GE) model of the economies of communities surrounding the PA. The GE model captures price as well as quantity effects, unlike fixed-price multiplier models typically used to study economic impacts of tourists. In our GE model, prices of goods traded with markets outside the local economy are fixed, but prices of non-tradable factors and goods (including labor and most services) are endogenous, determined by the interaction of local supply and demand under conventional market-closure constraints. We use the GE model to simulate the local economy impact of tourists and tourist spending on household incomes and the production of goods, retail sales, and services (S2-S7 Tables in S1 Appendix provide details on goods, factors, households, variables, parameters, and equations in the tourism GE model). Local economies were defined as the communities bordering the PA together with nearby towns within the PA’s sphere of economic influence, as determined by community members, tourism operators and the government (detailed definitions of the local economy at each site appear in S1 Text in S1 Appendix). A Monte Carlo method was used to construct 95% confidence intervals (CIs) around the simulation results [34, 35].

## Survey design for data inputs

Building the LEWIE tourism model required gathering data through surveys of tourists, lodges and resorts, local businesses, and local households. Our research team carried out primary surveys of households, businesses, tourists, and local lodges/hotels, which were then used for data analysis to feed as inputs in the LEWIE tourism model. The survey questionnaires for each location by survey types and the primary data are available as S1 Appendix. The methods used to design the surveys, the sampling procedure, and the information collected in the surveys are described in detail here.

**Fig 2:**
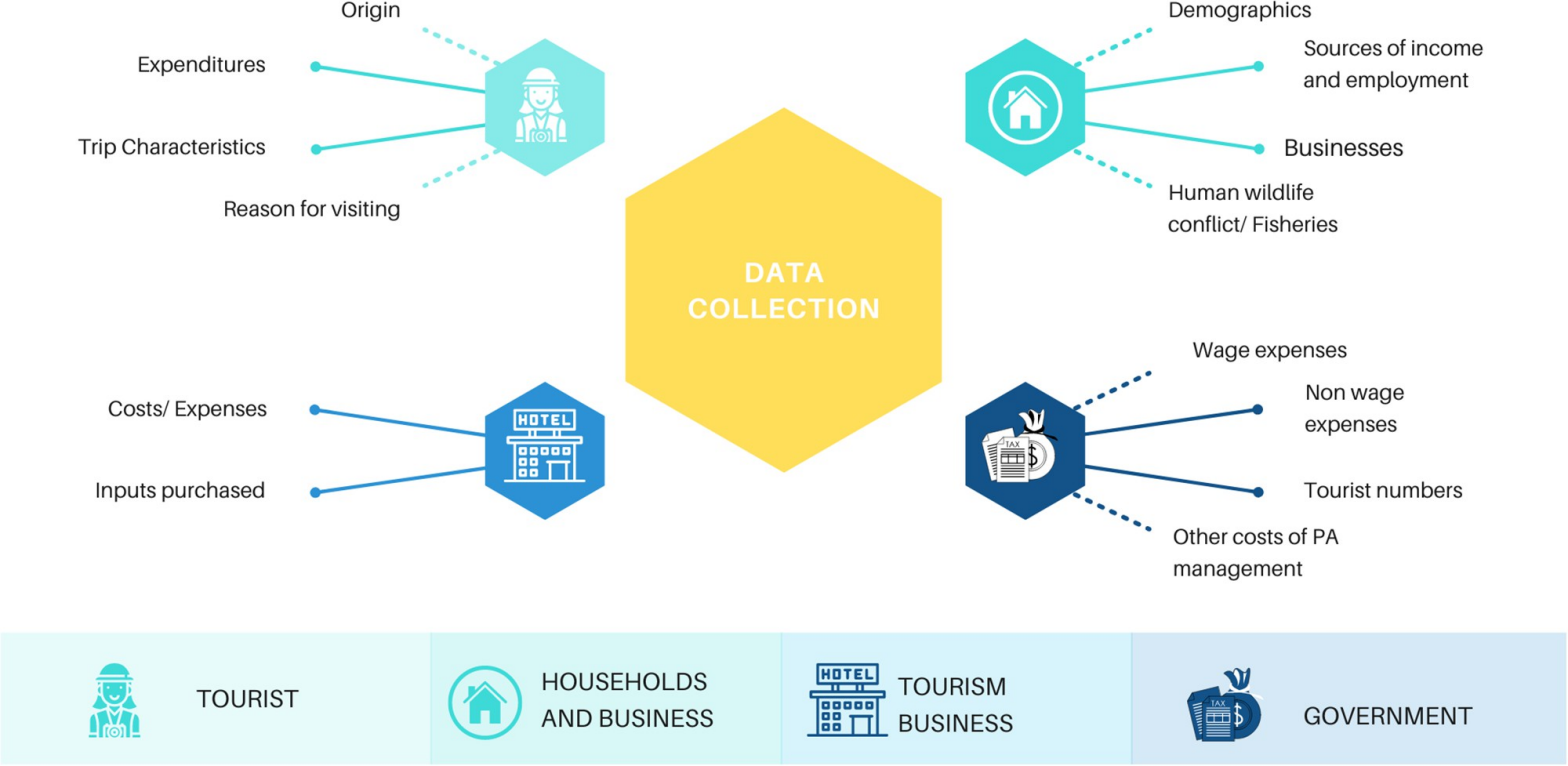
Survey data characteristics and different elements. Survey data on tourist, households, businesses, hotels, and lodges were collected in each PA. The data were used for econometric estimations, which were then used in the LEWIE model.

Household and business surveys gathered information on production, income, and expenditures, as well as the locations of transactions (i.e., whether inside or outside the local economy) (Fig 2). The household and local business surveys were programmed onto tablets using the Open Data Kit (ODK) platform for Android. Tourist survey information was collected through questionnaires implemented by the country (as in the case of Fiji’s International Visitor Survey), a tourism operator (in Zambia, Proflight Zambia made the questionnaire available to passengers on its return flights from the PAs to the capital, Lusaka), or secondary sources in the case of Brazil, where due to timing, COVID-19 travel restrictions prevented the collection of data from tourists, though not from households or businesses (sources included SEBRAE [36] and Statista [37, 38]). The collection of secondary data and the methods used to analyze them comply with the terms and conditions of the external data sources.

For the household and business surveys, communities in the area constituting the local economy were randomly selected from a master list. In each sampled village, roughly 45–55 households were randomly selected to be surveyed using an every-nth household sampling strategy based on the size and geographical dispersion of the community. The household survey included a module designed to gather information about businesses, and this was administered to households with businesses. Additional businesses in the villages and nearby market towns were surveyed to supplement the household business sample, using a business questionnaire with the same questions as in the household business module. Lacking access to a master list of businesses, all small businesses evident in each surveyed village were approached (villages typically had only a few businesses). In the neighboring market towns, an every-other-business approach was adopted for surveying. As in the household surveys, owner-operator participation in the business surveys was voluntary, with participation rates close to 100%. Government expenditure on PAs, wage and non-wage, was obtained from relevant government offices.

Random samples of 80–369 poor and 80–510 non-poor households per protected area were selected using an every-nth sampling strategy based on the size and geographical dispersion of communities. The household samples included 3.8% to 83% poor households at each site. Most households around the terrestrial PAs engaged in crop production. 50–80% of households at marine PAs were involved in fishing, and the scale of crop production was low compared with terrestrial PAs. About 24–36% of the sampled households owned and operated small businesses such as grocery shops, restaurants, etc. Survey data on 77–9,707 tourists were gathered through questionnaires administered by the country (in Fiji), by a tourism operator (in Zambia), or at hotels in which tourists stayed (in Brazil and Nepal). The households were classified into poor and non-poor groups. In the case of Fiji’s Mamanucas Islands, island households constituted an additional household group. Tourism business data were collected through interviews with lodge owners and tourism operators.

The local-economy wide modeling and surveys were approved by University of California Davis Institutional Review Board (IRB) protocol #303907–2. All participant consents were obtained before surveys orally. IRB #303907–2 approved oral consent upon participants being provided with a script information, which read, “*The Global Wildlife Program at The World Bank and the University of California, Davis, are carrying out a study of how countries can protect natural areas while creating economic benefits for their population, including those who live in and around parks. The information you provide will be extremely helpful in understanding the potential effects of nature tourism on local economies and improving policies related to tourism, conservation and sustained development. The results of this study will be shared at the UN2020 Convention on Biological Diversity. Your personal information will be treated with the highest degree of confidentiality. We will never share any of your personal information with ANYONE OUTSIDE of our research team. You may choose not to answer any questions you do not want to*.” Data are anonymized and no personal identifying information of participants are included in the manuscript. No data were collected on minors.

## Econometric estimation

Micro survey data provide initial values for all variables in the model (production inputs and outputs, household expenditures on goods and services). We assumed Cobb-Douglas production functions and Stone-Geary demands without subsistence minima. Activity-specific production functions were estimated for crops, livestock, fish, retail, services, and other production activities. We use Eq (1) for estimating the Cobb-Douglas production functions for each household group,

$$\begin{array}{c} y_{ik}=\beta_{0}+\beta_{1}land_{ik}+\beta_{1}labor_{ik}+\beta_{1}capital_{ik}+\beta_{1}inputs_{ik}+u_{ik} \end{array}$$ (1)

where *y*_*ik*_ is the value of output produced by household *i* in activity *k*, *land*_*ik*_ is the land, *labor*_*ik*_ is labor, *capital*_*ik*_ is capital, *input*_*ik*_ is input, respectively, used in activity *k* by household *i*, and *u*_*ik*_ is the idiosyncratic error term. All the variables in Eq (1) are inverse hyperbolic sine transformed and estimated using ordinary least squares (OLS). Household-specific expenditure functions were estimated using Eq (2) for these as well as goods purchased outside the local economy, transfers to and from other households, and formal and informal savings,

$$\begin{array}{c} expenditure\;in\;each\;category_{i}=\alpha_{0}+\alpha_{1}total\;expenditure_{i}+\xi_{i} \end{array}$$ (2)

where the expenditure in each category by household *i* are estimated as dependent variables of total expenditure. The contemporaneous errors associated with the dependent variables are correlated and thus a seemingly unrelated regression (SUR) that uses a feasible generalized least squares method is utilized that produces consistent and efficient estimators. These estimates yielded the model parameters for each household group and sector, as well as their standard errors (S10 Table in S1 Appendix).

## Local economy-wide impact evaluation tourism model

An additional tourist at each protected area generates economic activity in the local economy by stimulating local demand for goods and services, either directly (as when tourists buy goods and services from local businesses and households) or indirectly (as when lodges pay wages to local households or source goods from local businesses that in turn spend this income on locally supplied goods and services) (see Fig 1). Following a rich literature on agricultural household modeling [39, 40], we constructed separate micro-economic models of households around each PA using regression analysis of micro-survey data. The household models were nested within a GE model of each local economy [34]. The initial values of variables and parameters from econometric estimations interface with the GAMS (Generalized Algebraic Modeling System) software used to program the LEWIE model. S2-S7 Tables in S1 Appendix summarize the sets, accounts, variables, parameters, equation definitions, and equations in the model.

The model equations include production and input demand functions; expenditure functions for each household group and tourist; and local market-clearing conditions, which determine prices for nontradables or, for tradables with exogenous prices, net trade with outside markets. For each good and factor, closure rules determine where markets clear and how prices and wages are determined. A challenge in general-equilibrium modeling is that we usually do not know exactly where prices are determined. We assume that household and business capital and endowment of land are fixed and neither capital nor land can be reallocated between activities. This is a reasonable assumption in the short-run since for example crop cultivation implements are of little use in livestock or services activities, especially when markets are thin. Labor is tradable within the local economies, with endogenous local wages. Labor supply is likely to be elastic in and around the PAs, which are typically characterized by high rates of un- and under-employment. Labor supply elasticities cannot be estimated with available data, and we assumed a nearly perfectly elastic labor supply (= 100).

Because the model parameters are econometric estimates from micro-data, Monte Carlo methods can be used to test significance and construct confidence intervals around the simulated outcomes [34]. We conducted 500 iterations of the simulations at each PA by making an equivalent number of random draws from all model parameter distributions. GE modeling requires judgements, based on the survey data, about where and how prices are determined (i.e., market closure, which is not known with certainty), as well as model structure. Sensitivity analysis, combined with the Monte Carlo method described above, was used to test the robustness of simulated impacts to market-closure assumptions.

There is no way to know the true counterfactual of these local economies without the existence of the PA, but simulations using these models model offer the best approximation available. The impact of protected area tourism on a local economy is estimated in two steps. First, the impact of *an additional tourist* on the local economy is simulated together with the real-income multiplier of an additional dollar of tourist spending, as shown in Table 1. Second, the *total* impact is estimated by multiplying the per-tourist estimate by the number of tourists (see S1 Fig in S1 Appendix). The model was also used to understand the economic impacts of government policies and shocks to the economy, including the economic fallout of the COVID-19 pandemic, and the full economic cost of human-wildlife conflict at terrestrial PAs. (Human-wildlife conflict is not a major concern at the marine PAs).

**Table 1:** Impact of an additional tourist in the five protected areas.

*Impacts (in US$) per additional tourist*	Terrestrial Protected Areas			Marine Protected Areas	
	Zambia		Nepal	Brazil	Fiji
	Lower Zambezi	South Luangwa	Chitwan National Pk.	Abrolhos Marine Pk.	Mamanuca Islands
**Real income (Inflation-adjusted)**	1,355	1,045	169	357	2,400
	[1239,1471]	[944,1146]	[158,180]	[270,444]	[2270,2522]
One Tourist Spending	744	682	95	205	1,311
*To household groups*:					
Poor	737	913	21	61	544
Non-poor	618	132	148	296	1,652
Island	-	-	-	-	204
*Production by sector*:					
Crop	121	245	2	11	82
Livestock	80	67	2	18	139
Fish	-	-	-	11	99
Retail	228	614	75	197	1231
Services	414	288	42	130	701
Hotel	395	386	14	45	153

The numbers in the table are the impacts of an additional tourist in the local economy of each of the PA. 95% confidence intervals on total real-income impacts (in square brackets) were constructed by making random draws from all parameter distributions (S8-S9 Tables in S1 Appendix), recalibrating the base model, and repeating each simulation 1000 times. The dollar values are calculates using the following exchange rates: 1 Zambian kwacha = US$0.076; 1 Nepalese Rupee = US$0.0088; 1 Brazilian Real = US$0.19; 1 Fijian Dollar = US$0.44.

## Results and discussion

PAs attract tourists who spend money on a variety of tourism-related goods and services. Tourists pay park entrance fees, spend money at lodges and partake in tourism activities such as game drives, walking safaris, scuba-diving, and snorkeling offered by lodges or other tourism service providers. Tourists sometimes purchase goods and services directly from local businesses and households. A tourism impact analysis based on tourist expenditures would stop here and capture only a fraction of the impact on local economies. However, as tourism activities expand, they generate indirect impacts on local economies through production linkages. For example, tourist spending stimulates lodges’ and restaurants’ demands for intermediate inputs (goods and services) from local farms and businesses. Intermediate demands create positive linkage effects on the production side of the economy. An input-output (IO) analysis would stop here, capturing direct and indirect effects through production linkages.

A general equilibrium (GE) model captures these as well as income and consumption linkages. Production activities stimulated directly or indirectly by tourism pay incomes in the form of wages and profits. This income flows into local households, which in turn purchase goods and services inside and outside the local economy. As local activities expand to meet household demands, they unleash new rounds of increased production, intermediate input demand, income, and household expenditures. Successive rounds of impacts diminish in size, because some income leaks out of the local economy through trade. The total (direct and indirect) effects of PA tourism eventually converge to a local real income multiplier, defined as the change in household real income per unit of cash that PA tourists inject into the economy.

**Fig 3:**
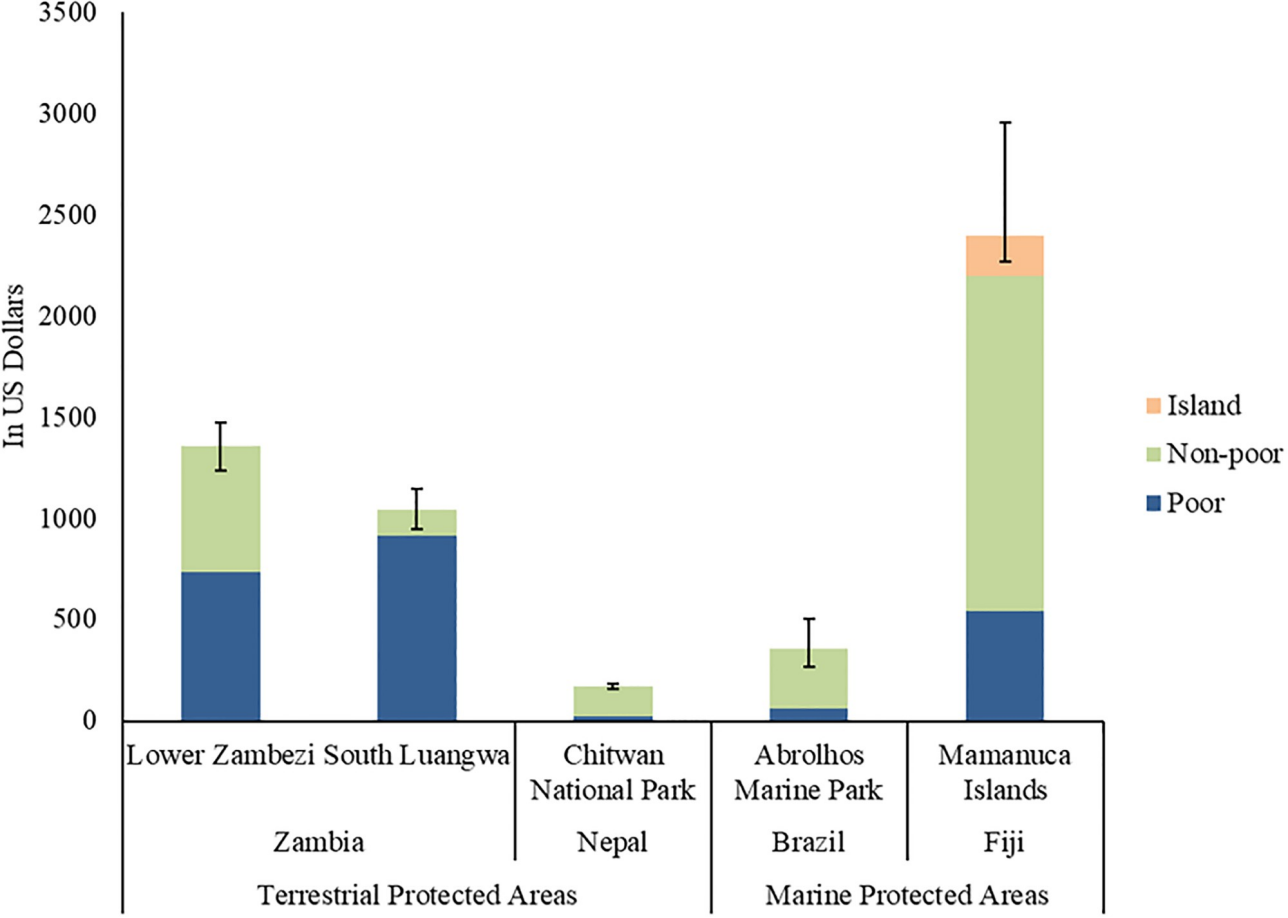
Impact of an additional tourist on real (inflation-adjusted) incomes in protected areas. The stacked vertical bars indicate the real (inflation-adjusted) impact of an additional tourist on total income in the local economy near the PA. The vertical line on each bar shows the confidence interval around this local economy impact. The blue and green colors decompose the total impacts by households’ poverty status.

Our GE simulations reveal that an additional tourist increases total real (inflation-adjusted) incomes by US$1,355 [CI: 1239, 1471] in and around Lower Zambezi National Park and US$1,045 [CI: 944, 1146] at South Luangwa National Park in Zambia; US$169 [CI: 158, 180] at Chitwan National Park in Nepal; US$357 [CI: 270, 444] at Abrolhos Marine Park in Brazil; and, US$2,400 [CI: 2270, 2522] in the Mamanuca Islands in Fiji (Table 1). At each site, we estimated real-income changes for poor and non-poor households (and “island households” in Fiji; see Fig 3), defined using national poverty lines and baseline incomes calculated from the survey data. Poor households receive 54% of the total real-income gain from tourism to Zambia’s Lower Zambezi National Park and 87% at South Luangwa National Park. Non-poor households capture a larger share of tourism benefits at the PAs in Nepal, Brazil, and Fiji. Real-income impacts of an additional tourist are substantial relative to local per-capita incomes. They represent 501–513% of average annual per-capita income around the two Zambia sites, 35% at Chitwan, 54% at Abrolhos, and 241% in the Mamanuca Islands.

The local income effects depend on how much money tourists spend, and this varies significantly among the PAs. Tourist expenditures are higher at sites in Zambia and Fiji: US$744 at Lower Zambezi, US$682 at South Luangwa, and US$1,311 in Fiji’s Mamanuca Islands. In contrast, spending per tourist is just over US$200 at the Brazil site and under US$100 in Nepal (Table 1). These differences reflect the type of tourism or tourists who visit PAs in the four countries. Sites in Fiji and Zambia draw high-value international tourists, Chitwan attracts low-value international and national tourists, and the Brazilian site draws mid-value domestic tourism. The total impact of tourism in protected areas on the local economy is the largest in Fiji, owing to both the high amount of tourist spending per day and the large volume of tourists. The two parks in Zambia also see high tourist spending from activities like guided safaris and hunting, but the parks have relatively few tourists due to a lack of connectivity. Nepal, on the other hand, generates a significant amount of revenue despite low spending per tourist, due to the sheer volume of park visitors. Large economic benefits from tourism around Nepal’s Chitwan National Park may come with trade-offs in terms of sustainability. If high volumes of tourists degrade the natural environment, fewer may visit in the future.

Multipliers per dollar spent by tourists facilitate comparisons across diverse PAs. Each additional dollar spent by a tourist creates real-income gains of $1.53-$1.82 at the terrestrial PA sites and $1.74-$1.83 at the marine PAs (Table 2). Poor households gain more than non-poor households at the two Zambia sites. Real-income multipliers are 1.34 for poor to 0.19 for non-poor households at South Luangwa National Park and 0.99 for poor and 0.83 for non-poor households at Lower Zambezi National Park. In contrast, non-poor households benefit more from an additional dollar of tourist at the PAs in Brazil, Fiji, and Nepal. The heterogenous impacts of tourist spending on real incomes of poor and non-poor households reflect the ways in which different household groups interact with activities that are affected directly or indirectly by tourism.

**Table 2:** Multiplier Effects of $1 of spending by tourists at protected areas.

*Multiplier per additional tourist*	Terrestrial Protected Areas			Marine Protected Areas	
	Zambia		Nepal	Brazil	Fiji
	Lower Zambezi	South Luangwa	Chitwan National Pk.	Abrolhos Marine Pk.	Mamanuca Islands
**Real income (Inflation-adjusted) multiplier**	1.82	1.53	1.78	1.74	1.83
*Multipliers disaggregated by household groups*:					
Poor	0.99	1.34	0.22	0.30	0.42
Non-poor	0.83	0.19	1.56	1.44	1.26
Island	-	-	-	-	0.15
**Production multiplier effects (by activities)**					
Crop	0.15	0.02	0.02	0.06	0.06
Livestock	0.10	0.01	0.03	0.09	0.11
Fish	-	-	-	0.05	0.08
Retail	0.29	0.03	0.78	0.97	0.94
Services	0.52	0.02	0.44	0.64	0.53
Hotel	0.50	0.00	0.15	0.22	0.12

The 95-percent confidence intervals around total real income multipliers are: Lower Zambezi [1.67, 1.96], South Luangwa [1.39, 1.67], Chitwan National Park [1.67, 1.91], Abrolhos Marine Park [1.31, 2.46], and Mamanuca Islands [1.74, 2.27]. Results were obtained by simulating a $1 increase in expenditure of a tourist in each protected area. The disaggregated impacts by household groups in each site add up to the total real income multiplier.

An additional dollar of tourist spending stimulates diverse local activities, including crop, livestock, and fish (at marine PAs) production, local retail, services, and hotels. Tables 1 and 2 report production impacts across diverse activities. The largest impacts of an additional tourist are on retail activities (US$75-US$1,231], most of which are family run shops and stores. This is where households spend the largest share of their incomes (about 30–70%, see S10 Table in S1 Appendix). Gross revenue from an additional tourist increases by US$42 to US$414 in services and US$14 to US$395 in hotels. There are positive impacts on crop and livestock production at terrestrial PAs and fish production at the marine PAs. Table 2 presents production multipliers, i.e., impacts on production per each additional dollar of tourist spending.

Just as tourism can have positive multiplier effects on local incomes, a loss of tourism has the opposite effect. Negative shocks produce negative local income multipliers. We estimate the impacts per month of lost tourism due to the COVID-19 pandemic at each of the five sites (see S2 Fig in S1 Appendix). These losses are felt most strongly by households that normally benefit directly from tourism. Around the Zambia parks, poor households suffer the greatest economic losses, while in Nepal, Fiji, and Brazil losses are larger for non-poor households.

Terrestrial PAs often experience human-wildlife conflicts that result in crop losses for households adjacent to parks. These crop production shocks, in turn, transmit negative ripple effects through local economies. The survey data already reflect losses from human-wildlife conflicts. Thus, we used a counterfactual of no human-wildlife conflict to simulate local-economy impacts of these losses. Specifically, the counterfactual simulation returns lost crops to households while simultaneously taking away the income that households received as compensation for the loss (if applicable). The total loss to the local economy is the negative of the real-income outcome from the counterfactual. Surveyed households reported that wildlife incursions onto farms caused crop losses of nearly 14% at Lower Zambezi and 11% at South Luangwa National Parks in Zambia and 9% around Chitwan National Park in Nepal. The resulting total annual real income losses are US$1.2–2.9 million. They are substantially larger for poor households in Zambia and non-poor households in Nepal (see S1 Table in S1 Appendix).

Annual impacts of tourism on real income at the five sites range from US$2.9 million to US$237.6 million. These estimates exclude the revenue tourism creates for governments, including park visitor and concession fees, as well as government use of these funds and how that might impact communities close to PAs. Because of this, our simulations are likely to underestimate the total economic impacts of PA tourism. Despite this, the findings from this study make a compelling case for governments to promote sustainable and inclusive tourism to PAs as part of a post-pandemic recovery strategy that fosters economic development while conserving biodiversity.

## Conclusions

This study provides estimates of the economic impacts of nature-based tourism on communities near PAs. Our simulations using data from five PAs in Brazil, Fiji, Nepal, and Zambia reveal that PA tourism can stimulate local economic development, raise incomes, and create employment for poor and non-poor households, including households that are not directly linked to tourism activities. This suggests that there is potential for PAs to contribute to development goals while maintaining a country’s rich biodiversity asset base. Findings from this research challenge perceptions of inherent tradeoffs between conserving biodiversity and promoting economic development and poverty alleviation. At the same time, local economies that depend on PA tourism (like any source of external income) are also vulnerable to tourism shocks. The COVID-19 pandemic and curtailment of international and domestic tourism appear to have had a disproportionately large negative impact on incomes in communities near PAs. Fully documenting economic benefits is a crucial step in making biodiversity conservation part of economic development and recovery plans and increasing funding for PAs, which appears to be the most robust predictor of successful ecological outcomes.

From a policy point of view, while countries are drawing some of these benefits from nature-based tourism, there is even greater potential for protected areas to contribute to development goals while maintaining a country’s rich biodiversity asset base. Our paper provides a framework to increase the triple bottom line benefits from protected areas by protecting natural assets, growing and diversifying the tourism business, sharing benefits with local communities, and making efforts towards a green recovery that is relevant and critical to development and poverty reduction.

By estimating both the direct and indirect impacts of tourism our paper provides a more complete picture of tourism’s linkages with communities around protected areas. However, the estimates provided are only a conservative estimate of the true impacts of protected area investment. Critical to this point, the GE model utilized in the paper is not dynamic, thus fluid factors like fishery stocks, which are critical to marine protected areas, are not considered. Scaling the LEWIE methodology to fit additional needs can provide further evidence of the benefits of investment in protected areas. Other unmeasured impacts also likely exist, for example, through the protection of ecosystem services.

Our study also does not estimate the costs of the adverse environmental impacts or externalities from tourists visiting protected areas, however, it recognizes that such activities could degrade the asset. Attribution poses another limitation in conducting the type of study presented here. Terrestrial PAs are often located far from cities, giving a clear purpose (wildlife, cultural heritage, geotourism) to tourists’ ventures to the area. In marine PAs, however, the motivations of tourists are less direct because visitors are also drawn to the areas by coastal beaches, regardless of whether the marine resources are protected. Because of this, the evidence that creating marine PAs will increase the number of tourists is lacking. Adding further data tailored to these cases and further tailoring these to the unique contexts of terrestrial and marine PAs will help to overcome this issue. Finally, the PAs studied here are not representative of the national level of their countries, nor are they indicative of how all parks in their given country function. Scaling this methodology to cover more parks and more contexts in future studies can help to overcome this issue.

Biodiversity conservation creates economic costs as well as benefits, including losses from human-wildlife conflict. Our simulations find that these costs are small compared with the total economic benefits of tourism to PAs. However, households that bear the costs of biodiversity conservation are not necessarily the ones that benefit, directly or indirectly, from nature-based tourism. Creating economic incentives to conserve biodiversity includes measures to compensate households and communities from adverse economic impacts including human-wildlife conflicts.

## Supporting information

S1 Appendix (PDF)

S1 File (ZIP)
