# Supplementary material for: Economic impact of nature-based tourism
Source: PLoS One. 2023 Apr 12;18(4):e0282912. doi: 10.1371/journal.pone.0282912 (PMC10096494; doi:10.1371/journal.pone.0282912)
Supplement: S1 Appendix — (PDF) [file pone.0282912.s001.pdf]

Supporting Information for
Economic Impact of Nature-based Tourism

Anubhab Gupta*, Heng Zhu, Hasita Bhammar, Elisabeth Earley, Mateusz Filipski, Urvashi Narain, Phoebe Spencer, Edward Whitney, and J. Edward Taylor

*Corresponding author. Email: anubhab@vt.edu

This PDF file includes:

Supplementary Text
Figs. S1 to S2
Tables S1 to S10

Other Supplementary Materials for this manuscript include the following:

LEWIE Model for Brazil Abrolhos Marine Park (GAMS text file)
LEWIE Model for Fiji Mamanuca Islands (GAMS text file)
LEWIE Model for Nepal Chitwan National Park (GAMS text file)
LEWIE Model for Zambia Lower Zambezi South Luangwa (GAMS text file)

Data Input Sheets (in EXCEL)
1. base_inputsheet_wb_Brazil_v9
2. base_inputsheet_wb_Fiji_v13
3. base_inputsheet_wb_Nepal_v16
4. base_inpusheet_ wb_Zambia_v15

Survey Instruments and Primary Data (in EXCEL)
1. Household Surveys and Data (Brazil, Fiji, Nepal, Zambia)
2. Business Surveys and Data (Brazil, Fiji, Nepal, Zambia)
3. Tourist Surveys and Data (Brazil, Fiji, Nepal, Zambia)
4. Hotel/Lodge Surveys and Data (Brazil, Fiji, Nepal, Zambia)

Supplementary Text

Detailed Definition of Local Economy

A "local economy" is defined for this study as the economy of communities located near protected areas (PAs). To be effective, management plans for protected areas (PAs) incentivize surrounding communities to support conservation. For the purpose of this study, the "local economy" of PAs is defined by the communities that lie within the protected area's sphere of economic influence as determined by community members, tourism operators and the government. The local economy for each PA is defined below. Because village households and businesses routinely visited a nearby market town to purchase goods and services, market towns nearest to each park were also included as part of the local economy for the studies.

- *Lower Zambezi National Park (Zambia):* Chiawa Game Management Area (GMA) and the market town of Chirunda constitute the local economy for Lower Zambezi NP. Lower Zambezi NP was established in 1983 and designated as IUCN Category II. It has 6 lodges and an airstrip inside the park.
- *South Luangwa National Park (Zambia):* The local economy of South Luangwa NP includes the Upper and Lower Lupande GMA and the market town of Chipata. Established in 1972 and designated as IUCN Category II, South Luangwa NP has 21 lodges inside the park and the Mfuwe International Airport is at the point of entry.
- *Chitwan National Park (Nepal):* Three national municipalities of Bharatpur, Khairahani, and Ratnanagar constitute the local economy of Chitwan NP. The Chitwan NP, designated as IUCN Category II, was declared as a UNESCO World Heritage Site in 1984.
- *Abrolhos Marine National Park (Brazil):* This PA is a mostly uninhabited archipelago off the southern coast of the the state of Bahia in northeastern Brazil. The Abrolhos region including the coastline (extending from Nova Viçosa in the south to the south-west of Caravelas and to Prado in the North) opposite Abrolhos Marine National Park constitutes the local economy for this study. Established in 1983, Abrolhos Marine NP is designated as IUCN Category II and has the largest whale nursery in the South Atlantic Ocean. Whale watching is the biggest attraction for tourists visiting the Abrolhos Marine NP every year.
- *Mamanuca Islands (Fiji):* Three Mamanuca islands, Tavarua, Navini, and Malolo, together with the mainland opposite them in the western coastal region of Nadroga-Navosa province, including its main city, Nadi, constitute the local economy for this study. These islands are a popular destination for tourists due to their pristine waters and coral reefs.

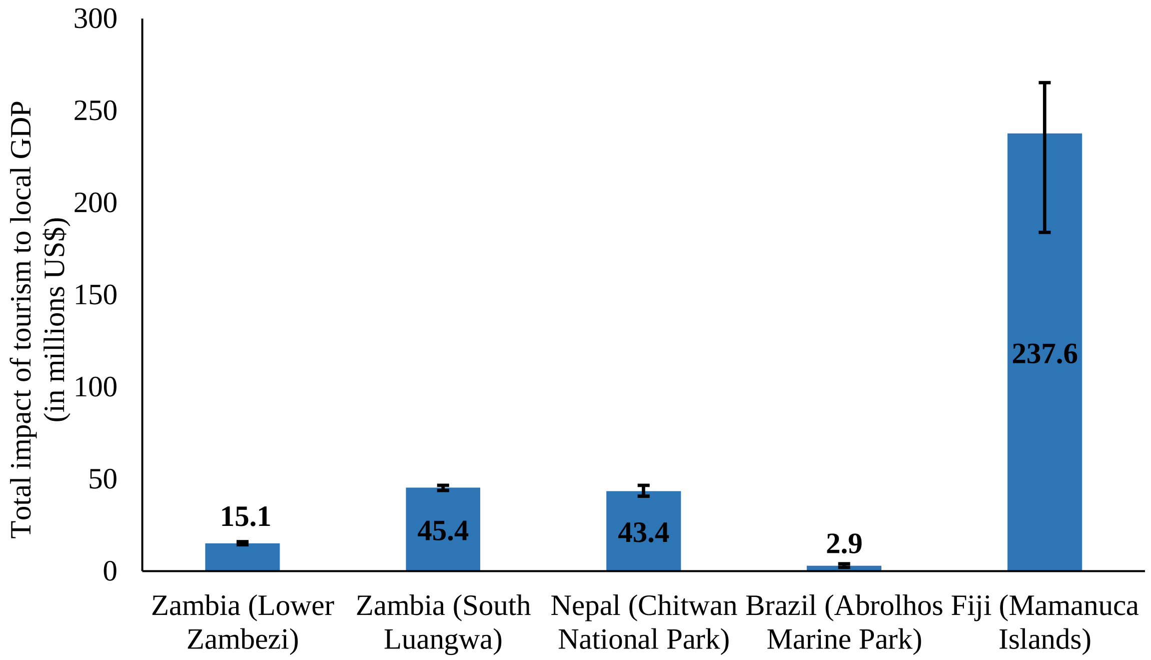

Figure S1: Annual real (Inflation-adjusted) impact of Protected Area tourism on local GDP (in millions of US$)

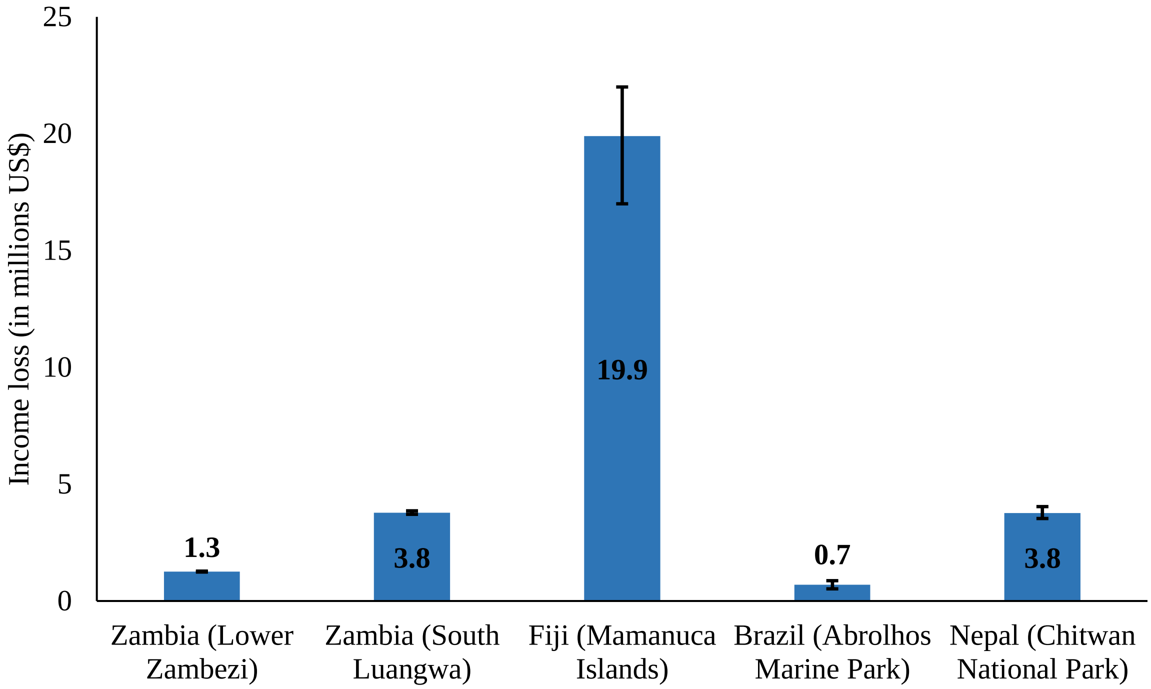

Figure S2: Monthly real (Inflation-adjusted) income loss in Protected Area from no tourism due to the COVID-19 pandemic (in millions of US$)

Table S1: Simulated Impacts of Annual Loss due to Human-Wildlife Conflict in Three Terrestrial Protected Areas

**Annual Loss (In US$)**	**Zambia**		**Nepal**
	Lower Zambezi	*South Luangwa*	*Chitwan National Park*
**Real income (Inflation-adjusted)**	1,798,571	1,238,969	2,946,158
By household groups			
Poor	1,122,494	1,024,838	206,007
Non-poor	676,077	214,131	2,846,888
Loss in Production Value			
Crop	1,119,384	979,156	1,513,543

The numbers are the simulated impacts of wildlife incursions onto farms that cause crop losses of almost 14 percent at Lower Zambezi National Park, 11 percent at South Luangwa National Park, and 9 percent at Chitwan National Park, respectively. The model uses harvest data reported at the time of the survey. The cost of human-wildlife conflict is therefore estimated as a counterfactual.

Table S2. Set, Subset and Mapping Names Used in Model Statement

SETS		Subsets	
g	commodities	gtv	Goods locally tradable
f	Factors	gtz	Goods traded in outside markets
h or hh	households	gp	Locally produced goods
		gag	Agricultural goods
		gnag	Nonagricultural goods
v	Protected Area (PA) clusters	fk	Fixed factors
		ft	Locally tradable factors
Mappings		ftw	Factors traded in outside markets
maphv(h,v)	Mapping of households to their PA clusters	fpurch	Purchased variable inputs

Table S3. Commodities, Factors, Households, and PAs

**Commodities**	
Crop	Local crops: produced and consumed within the cluster
Livestock	Local livestock, produced and consumed within the cluster
Fish	Local fish, harvested and consumed within the cluster (only in Marine Terrestrial PAs)
Retail	Local retailers in the cluster
Services	Local Services in the cluster
Hotel	Hotels for Tourists
Outside	Any commodity purchased outside the local economy
**Factors**	
Labor	Labor (family and hired receiving wage in cash or kind)
Land	Land
Capital	Capital
Input	Purchased inputs
**Households**	
Poor	Poor households in PAs
Non-poor	Non-poor households in PAs
Island	Households in Mamanuca islands (only in Fiji Marine PA)
**PAs**	
	Terrestrial<br>Lower Zambezi National Park & South Luangwa National Park (Zambia)<br>Chitwan National Park (Nepal)<br>*Marine*<br>Abrolhos Marine Park (Brazil)<br>Mamanuca Islands (Fiji)

Table S4. Variable Names Used in Model Statement

VARIABLES			
**Values**		**Consumption and income**	
PV(g,v)	price of a good at the cluster level	QC(g,h)	quantity of g consumed by h
PZ(g)	price of a good at the local-economy level	Y(h)	nominal household income
PH(g,h)	price as seen by household h (=PV or PZ)	RY(h)	real household income
PVA(g,h)	price of value added net of intermediate inputs	CPI(h)	consumer price index
R(g,f,h)	rent for fixed factors	TROUT(h)	transfers given by a household of others
WV(f,v)	wage at the cluster level	SAV(h)	household savings
WZ(f)	wage at the regional level	EXPROC(h)	household expenditures out of the region
**Production**		**Trade**	
QP(g,h)	quantity produced of a good by a household	HMS(g,h)	household marketed surplus of good g
FD(g,f,h)	factor demand of f in production of g	VMS(g,v)	cluster marketed surplus of good g
ID(g,gg,h)	intermediate demand for production of g	ZMS(g)	Regional marketed surplus of a good
QVA(g,h)	quantity of value added created	HFMS(f,h)	factor marketed surplus from the household
HFD(f,h)	factor demand in the household	VFMS(f,v)	factor marketed surplus out of the cluster
HFSUP(f,h)	labor supply from the household (elastic endowment)	ZFMS(f)	factor marketed surplus out of the region

Table S5. Parameter Names Used in Model Statement (GAMS)

PARAMETERS<br>Production		 Consumption	
a(g,h)	Shift parameter in CD production function	alpha(g,h)	consumption share parameters in the LES
beta(g,f,h)	Factor share parameters (CD exponents)	cmin(g,h)	minimal consumption in the LES
vash(g,h)	Value-added share of output	exinc(h)	exogenous income of household
idsh(gg,g,h)	Intermediate input share	vmsfix(g,v)	fixed marketed surplus at the cluster level
fixfac(g,f,h)	Fixed factor endowments	Transfers	
vfmsfix(f,v)	Factors fixed at the local level (family, hired labor)	troutsh(h)	share of transfers in household expenditures
		exprocsh(h)	share of expenditures outside 10 km radius of camp
endow(f,h)	Household factor endowments	savsh(h)	share of income saved
hfsupzero(f,h)	Initial labor supply	trinsh(h)	share of total transfers received by a given household
hfsupel(f,h)	Factor supply elasticity	For Experiments	
		transfer(h)	WFP transfer to household
pibudget(g,h)	Liquidity constraint on inputs	hfsnewref(ft,h)	Refugee labor supply
pibsh(g,h)	Share of pibudget to good g	packsold(g)	In-kind transfer sold on market

Table S6. Equation Definitions

Equation Name	Description
*** prices**	
EQ_PVA(g,h)	price value added equation
EQ_PH(g,h)	market price as seen from household h
*** production**	
EQ_FDCOBB(g,f,h)	factor demands Cobb-Douglas
EQ_FDPURCH(g,f,h)	factor demands for purchased inputs - constrained or not
EQ_QVACOBB(g,h)	quantity VA produced Cobb-Douglas
EQ_QP(g,h)	quantity produced from QVA and ID
EQ_ID(gg,g,h)	quantity of ID needed for QP
*** consumption**	
EQ_QC(g,h)	quantity consumed
*** income**	
EQ_Y(h)	full income constraint for the household
EQ_CPI(h)	consumer price index equation
EQ_RY(h)	real household income equation
*** transfers**	
EQ_TRIN(h)	inter household transfers in (received)
EQ_TROUT(h)	interhousehold transfers out (given)
*** exogenous expenditures**	
EQ_SAV(h)	savings (exogenous rate)
EQ_EXPROC(h)	expenditures outside of the cluster (exogenous rate)
*** goods market clearing**	
EQ_HMKT(g,h)	qty clearing in each household
EQ_VMKT(g,v)	market clearing in the cluster
EQ_ZMKT(g)	market clearing in the region
EQ_VMKTfix(g,v)	price definition in the cluster
EQ_ZMKTfix(g)	price definition in the region
*** factor market clearing**	
EQ_HFD(f,h)	total household demand for a given factor
EQ_FCSTR(g,f,h)	fixed factors constraint
EQ_HFSUP(f,h)	household elastic supply
EQ_HFMKT(f,h)	tradable factor clearing in the household
EQ_VFMKT(f,v)	tradable factors clearing in the village
EQ_ZFMKT(f)	tradable factor clearing in the region
EQ_VFMKTfix(f,v)	wage determination for tradable factors clearing in the village
EQ_ZFMKTfix(f)	wage determination for tradable factors clearing in the region
*** In case of nlp solve**	
EQ_USELESS	trick to make GAMS think it's maximizing something

Table S7. Equations in the Model

Name	Equation
1) HOUSEHOLD EQUATIONS	
Price Block	
EQ_PH(g,h)..	$PH_{g,h} = [PZ_g]_{g\in gtz \cup gtw} + \left[\sum_{v\|maphv(h,v)} PV_{g,v}\right]_{g\in gtv}$
EQ_PVA(g,h)..	$PVA_{g,h} = PH_{g,h} - \sum_{ga} idsh_{ga,g,h} \times PH_{ga,h}$
Production Block	
EQ_QVACOBB(g,h)..	$QVA_{g,h} = a_{g,h} \times \prod_{f} (FD_{g,f,h})^{\beta_{g,f,h}}$
EQ_FDCOBB(g,f,h)	$[R_{g,f,h}]_{f\in fk} + [WZ_f]_{f\in ftz} + \left[\sum_{v\|maphv(h,v)} WV_{f,v}\right]_{f\in ftv} = \frac{PVA_{g,h} \times QP_{g,h} \times \beta_{g,f,h}}{FD_{g,f,h}}$
EQ_QP(g,h)	$QP_{g,h} = QVA_{g,h}/vash_{g,h}$
EQ_ID(gg,g,h)..	$ID_{ga,g,h} = QP_{g,h} \times idsh_{ga,g,h}$
Consumption and income block	
EQ_QC(g,h)..	$QC_{g,h} = \frac{\alpha_{g,h}}{PH_{g,h}} \times \left(Y_h - TROUT_h - SAV_h - EXPROC_h - \sum_{ga} PH_{ga,h} \times cmin_{ga,h}\right) + cmin_{g,h}$
EQ_Y(h)..	$Y_h = \sum_{g,fk} (R_{g,fk,h} \times FD_{g,fk,h}) + \sum_{g,ftz} WZ_{ftz} \times HFSUP_{ftz,h} + \sum_{ftv} \sum_{v\|maphv(h,v)} WV_{ftv,v} \times HFSUP_{ftv,h} + \sum_{ftw} WZ_{ftw} \times HFSUP_{ftw,h}$
EQ_TROUT(h)..	$TROUT_h = troutsh_h \times Y_h$
EQ_EXPROC(h)..	$EXPROC_h = exprocsh_h \times Y_h$
EQ_SAV(h)..	$SAV_h = savsh_h \times Y_h$
EQ_CPI(h)..	$CPI_h = \sum_{g} PH_{g,h} \times \alpha_{g,h}$
EQ_RY(h)..	$RY_h = \frac{Y_h}{CPI_h}$
2) MARKET CLOSURE:	

Market clearing block for commodities	
EQ_HMKT(g,h)..	$$HMS_{g,h} = QP_{g,h} - QC_{g,h} - \sum_{ga} ID_{g,ga,h}$$
EQ_VMKT(g,v)..	$$VMS_{g,v} = \sum_{h\|maphv(h,v)} HMS_{g,h}$$
EQ_ZMKT(g)..	$$ZMS_{g,v} = \sum_{v} VMS_{g,v}$$
EQ_VMKTfix(gtv,v)..	$$VMS_{gtv,v} = vmsfix_{gtv,v}$$
EQ_ZMKTfix(gtz)..	$$ZMS_{gtz} = zmsfix_{gtz}$$
Market clearing block for factors	
EQ_HFV(f,h)..	$$HFD_{f,h} = \sum_{g} FD_{g,f,h}$$
EQ_FCSTR(g,fk,h)..	$$FD_{g,fk,h} = fixfac_{g,fk,h}$$
EQ_HFMKT(ft,h)..	$HFMS_{ft,h} = HFSUP_{ft,h} - \sum_g FD_{g,ft,h}$
EQ_HFSUP(ft,h)..	$$HFSUP_{ft,h} = hfsup^{0}_{ft,h} * \left[\left[\sum_{d\|maphd(h,d)} (WD_{ft,d})^{\zeta_{ft,h}}\right]_{f\in ftd} + \left[(WZ_{ft,d})^{\zeta_{ft,h}}\right]_{f\in ftz\cup ftw}\right]$$
EQ_VFMKT(ft,v)..	$DFMS_{g,d} = \sum_{h\|maphd(h,d)} HFMS_{g,h}$
EQ_ZFMKT(ft)..	$$ZFMS_{ft} = \sum_{v} VFMS_{ft,v}$$
EQ_VFMKTFIX(ftv,v)..	$$VFMS_{ftd,d} = vfmsfix_{ftv,v}$$
EQ_ZFMKTFIX(ftz)..	$$ZFMS_{ftz} = zfmsfix_{ftz}$$
For simulations with a budget constraint	
EQ_FDCOBB(g,f,h)<br><br>(only for purchased factors)	$$FD_{g,f,h} \times WZ_{f} = pibudget_{g,h}$$

Table S8. Production Function Estimates and Standard Errors (Terrestrial PAs)

Production Activity	Parameter	Input	Lower Zambezi		South Luangwa		Chitwan National Park	
			Poor	Non-poor	Poor	Non-poor	Poor	Non-poor
Crop	Estimate	Land	0.39	0.39	0.49	0.49	0.64	0.59
		Labor	0.34	0.34	0.21	0.21	0.13	0.19
		Capital	0.06	0.06	0.06	0.06	0.16	0.14
		Input	0.21	0.21	0.23	0.23	0.07	0.08
	Standard Error	Land	0.07	0.07	0.12	0.12	0.07	0.07
		Labor	0.08	0.08	0.10	0.10	0.05	0.07
		Capital	0.02	0.02	0.00	0.00	0.04	0.09
		Input	0.06	0.06	0.23	0.23	0.04	0.03
	Shift Parameter		6.24	6.24	6.85	6.85	3.08	3.30
	se		0.31	0.31	0.29	0.29	0.39	0.56
Livestock	Estimate	Land	0.15	0.12	0.43	0.30	0.11	0.07
		Labor	0.07	0.11	0.08	0.08	0.15	0.23
		Capital	0.47	0.66	0.33	0.48	0.56	0.60
		Input	0.30	0.11	0.16	0.13	0.18	0.10
	Standard Error	Land	0.02	0.02	0.02	0.07	0.04	0.04
		Labor	0.01	0.02	0.01	0.02	0.04	0.06
		Capital	0.04	0.06	0.02	0.09	0.07	0.09
		Input	0.03	0.03	0.02	0.05	0.04	0.04
	Shift Parameter		4.55	2.87	5.91	4.97	2.98	2.59
	se		0.30	0.40	0.15	0.73	0.44	0.49
Retail	Estimate	Labor	0.47	0.47	0.44	0.44	0.75	0.75
		Capital	0.26	0.26	0.22	0.22	0.16	0.16
		Input	0.27	0.27	0.34	0.34	0.09	0.09
	Standard Error	Labor	0.07	0.07	0.07	0.07	0.05	0.05
		Capital	0.06	0.06	0.05	0.05	0.05	0.05
		Input	0.08	0.08	0.07	0.07	0.04	0.04
	Shift Parameter		3.68	3.68	3.49	3.49	6.66	6.66
	se		0.39	0.39	0.41	0.41	0.54	0.54
Services	Estimate	Labor	0.54	0.54	0.60	0.60	0.53	0.53
		Capital	0.22	0.22	0.29	0.29	0.40	0.40
		Input	0.15	0.15	0.10	0.10	0.07	0.07
	Standard Error	Labor	0.10	0.10	0.06	0.06	0.09	0.09
		Capital	0.08	0.08	0.06	0.06	0.08	0.08
		Input	0.11	0.11	0.08	0.08	0.05	0.05
	Shift Parameter		3.76	3.76	3.66	3.66	3.82	3.82
	se		0.61	0.61	0.30	0.30	0.85	0.85
Hotel	Estimate	Labor	0.48	0.48	0.44	0.44	0.81	0.81
		Capital	0.52	0.52	0.56	0.56	0.19	0.19
N			254	183	369	80	311	244

Table S9. Production Function Estimates and Standard Errors (Marine PAs)

Production Activity	Parameter	Input	Abrolhos Park		Mamanucas Islands		
			Poor	Non-poor	Poor	Non-poor	Islands
Crop	Estimate	Land	0.272	0.327	0.409	0.409	0.409
		Labor	0.190	0.337	0.409	0.409	0.409
		Capital	0.230	0.155	0.142	0.142	0.142
		Input	0.308	0.181	0.072	0.072	0.072
	Standard Error	Land	0.090	0.048	0.040	0.040	0.040
		Labor	0.088	0.070	0.041	0.041	0.041
		Capital	0.110	0.047	0.028	0.028	0.028
		Input	0.104	0.050	0.030	0.030	0.030
	Shift Parameter		3.624	2.422	1.833	1.833	1.833
	se		0.745	0.405	0.231	0.231	0.231
Livestock	Estimate	Land	0.110	0.110	0.144	0.144	0.158
		Labor	0.400	0.400	0.308	0.308	0.487
		Capital	0.250	0.250	0.416	0.416	0.220
		Input	0.240	0.240	0.131	0.131	0.136
	Standard Error	Land	0.040	0.040	0.033	0.033	0.087
		Labor	0.128	0.128	0.048	0.048	0.090
		Capital	0.074	0.074	0.059	0.059	0.114
		Input	0.121	0.121	0.032	0.032	0.051
	Shift Parameter		3.190	3.190	3.017	3.017	3.377
	se		0.645	0.645	0.334	0.334	0.578
Fish	Estimate	Labor	0.317	0.317	0.755	0.755	0.755
		Capital	0.596	0.596	0.109	0.109	0.109
		Input	0.087	0.087	0.136	0.136	0.136
	Standard Error	Labor	0.130	0.130	0.044	0.044	0.044
		Capital	0.143	0.143	0.025	0.025	0.025
		Input	0.072	0.072	0.053	0.053	0.053
	Shift Parameter		1.000	1.000	1.830	1.830	0.972
	se		0.000	0.000	0.129	0.129	0.200
Retail	Estimate	Labor	0.286	0.286	0.550	0.550	0.550
		Capital	0.271	0.271	0.250	0.250	0.250
		Input	0.443	0.443	0.200	0.200	0.200
	Standard Error	Labor	0.042	0.042	0.061	0.061	0.061
		Capital	0.051	0.051	0.069	0.069	0.069
		Input	0.058	0.058	0.052	0.052	0.052
	Shift Parameter		2.503	2.503	3.233	3.233	3.233
	se		0.328	0.328	0.384	0.384	0.384

Continued to next page…

Production Activity	Parameter	Input	Abrolhos Park		Mamanucas Islands		
Services	Estimate	Labor	0.356	0.356	0.529	0.529	0.529
		Capital	0.211	0.211	0.216	0.216	0.216
		Input	0.433	0.433	0.255	0.255	0.255
	Standard Error	Labor	0.056	0.056	0.049	0.049	0.049
		Capital	0.062	0.062	0.065	0.065	0.065
		Input	0.088	0.088	0.077	0.077	0.077
	Shift Parameter		2.881	2.881	3.480	3.480	3.480
	se		0.431	0.431	0.493	0.493	0.493
Hotel	Estimate	Labor	0.509	0.509	0.698	0.698	0.698
		Capital	0.491	0.491	0.302	0.302	0.302
N			80	510	322	75	122

Notes: Tables S9a and S9b present activity-specific Cobb-Douglas production function estimates and their respective standard errors for households (crop, livestock, and fish) and businesses (retail, services, and hotel). All production functions are estimated with constant returns to scale technology. The factors of production for crop and livestock in both terrestrial and marine PAs are land, labor, capital, and purchased inputs. In marine PAs, fish production takes place with labor, capital, and purchased inputs. For business activities in both terrestrial and marine PAs, factors of production are labor, capital, and purchased inputs. Hotel lodging and services are estimated using labor and capital as factors of production.

Table S10. Expenditure Function Parameter Estimates and Standard Errors

(a) Terrestrial Protected Areas

Parameter	Commodity	Lower Zambezi			South Luangwa			Chitwan National Park		
		Poor	Non-poor	Tourists	Poor	Non-poor	Tourists	Poor	Non-poor	Tourists
Estimate	Crop	0.14	0.07	-	0.11	0.12		0.12	0.04	-
	Livestock	0.08	0.10	-	0.06	0.11		0.09	0.02	-
	Retail	0.53	0.63	0.06	0.63	0.48	0.05	0.30	0.63	0.08
	Services	0.22	0.18	0.06	0.19	0.28	0.03	0.46	0.27	0.25
	Hotel	-	-	0.14	-	-	0.20	-	-	0.44
	Outside	0.02	0.02	0.74	0.00	0.00	0.72	0.04	0.04	0.22
Standard Error	Crop	0.02	0.03	-	0.01	0.02	-	0.06	0.00	-
	Livestock	0.01	0.02	-	0.01	0.01	-	0.05	0.00	-
	Retail	0.03	0.04	0.00	0.02	0.03	0.00	0.07	0.02	0.00
	Services	0.03	0.03	0.00	0.02	0.02	0.00	0.10	0.02	0.00
	Hotel	-	-	0.00	-	-	0.00	-	-	0.00
	Outside	-	-	0.00	-	-	0.00	-	-	0.00
N		254	183	226	369	80	226	311	244	77

(b) Marine Protected Areas

Parameter	Commodity	Alborhos Park			Mamanucas Islands			
		Poor	Non-poor	Tourists	Poor	Non-poor	Islands	Tourists
Estimate	Crop	0.08	0.03	-	0.03	0.03	0.06	-
	Livestock	0.06	0.05	-	0.04	0.11	0.07	-
	Fish	0.03	0.03	0.01	0.03	0.04	0.03	-
	Retail	0.43	0.54	0.10	0.71	0.52	0.47	0.18
	Services	0.34	0.31	0.11	0.18	0.28	0.35	0.38
	Hotel	-	-	0.62	-	-	-	0.36
	Outside	0.06	0.05	0.16	0.02	0.02	0.01	0.08
Standard Error	Crop	0.02	0.00	-	0.00	0.02	0.01	-
	Livestock	0.01	0.01	-	0.01	0.02	0.01	-
	Fish	0.01	0.01	0.00	0.01	0.02	0.01	
	Retail	0.03	0.03	0.00	0.02	0.05	0.02	0.10
	Services	0.03	0.02	0.00	0.02	0.04	0.02	0.22
	Hotel	-	-	0.00	-	-	-	0.20
	Outside	-	-	0.00	-	-	-	0.08
N		80	510	501	322	75	122	9707

Notes: Panels (a) and (b) of Table S10 present the expenditure function estimates with their standard errors for the poor and non-poor household groups as well as the tourists in terrestrial and marine PAs, respectively. The expenditure functions are estimated as seemingly unrelated regression (SUR) specifications. The parameter estimate for "Outside" commodity is estimated as a residual expenditure share, i.e., sum of all other expenditure shares subtracted from 1. The standard errors are rounded to two decimal points for clean presentation, and thus 0 standard error indicates very low value.
